# An Increased Circular RNA, circRNPS1, in T Cells of Ankylosing Spondylitis Patients Enhances the MEK/ERK Signaling and Inflammation Mediated by T Cell Activation via Stabilization of hnRNPK and VAV1

**DOI:** 10.3390/ijms27177550

**Published:** 2026-08-23

**Authors:** Hui-Chun Yu, Ning-Sheng Lai, Kuang-Yung Huang, Pin-Chen Chen, Ming-Chi Lu, Hsien-Bin Huang

**Affiliations:** 1 Department of Medical Research, Dalin Tzu Chi Hospital, Buddhist Tzu Chi Medical Foundation, Chiayi 62247, Taiwan; junvsusagi@gmail.com (H.-C.Y.); love19961215@gmail.com (P.-C.C.); 2 Division of Allergy, Immunology and Rheumatology, Department of Medicine, Dalin Tzu Chi Hospital, Buddhist Tzu Chi Medical Foundation, Chiayi 62247, Taiwan; q12015@tzuchi.com.tw (N.-S.L.); hky0919@yahoo.com.tw (K.-Y.H.); 3 School of Medicine, Tzu Chi University, Hualien 62102, Taiwan; 4 Department of Biomedical Sciences, National Chung Cheng University, Chiayi 62102, Taiwan

**Keywords:** ankylosing spondylitis, circRNA, circRNPS1, hnRNPK, MEK/ERK signaling, inflammatory cytokines

## Abstract

Ankylosing spondylitis (AS) is a chronic inflammatory disease strongly associated with human leukocyte antigen B27 (HLA-B27). However, the incomplete penetrance of HLA-B27 suggests that additional molecular mechanisms contribute to AS pathogenesis. Circular RNAs (circRNAs) have emerged as important regulators of immune responses, but their roles in AS pathogenesis remain incompletely understood. In this study, circRNA expression profiles were examined by high-throughput RNA sequencing and selected circRNAs were validated by quantitative RT-PCR in T cells from AS patients and healthy controls. Among the validated circRNAs, circRNPS1 was significantly up-regulated in T cells from AS patients. Overexpression of circRNPS1 in activated Jurkat cells increased *IL-2*, *IL-17A*, and *interferon-γ* expression and enhanced JAK2/STAT1 and MEK1/2-ERK signaling. RNA pull-down followed by proteomic analysis identified heterogeneous nuclear ribonucleoprotein K (hnRNPK) that binds to the back-splicing junction region of circRNPS1. CircRNPS1 increased the stability of hnRNPK and VAV1, whereas *hnRNPK* knockdown attenuated circRNPS1-mediated MEK1/2-ERK activation and inflammatory cytokine expression. Furthermore, an antisense RNA fragment targeting the back-splicing junction region of circRNPS1 suppressed *IL-2*, *IL-17A*, and *interferon-γ* expression in activated circRNPS1-overexpressing Jurkat cells and T cells from AS patients. Disrupting the interaction between circRNPS1 and hnRNPK therefore warrants further investigation as a potential therapeutic strategy for AS.

## 1. Introduction

Ankylosing spondylitis (AS) is an autoimmune disorder. The clinical characteristic of AS in the early stage is the long-term inflammation in sacroiliac joint, spine, and peripheral joints. New bone formation will be developed on sacroiliac joint and the axial joint of spine in some AS patients, in turn resulting in ankylosis and syndesmophytes [1,2,3]. The pathogenesis of AS is highly associated with the expression of human leukocyte antigen-B27 (HLA-B27), one of the MHC class I molecules [4,5]. More than 90% of AS patients express HLA-B27. However, only around 1–2% of all HLA-B27-positive people develop AS, suggesting that other genetic and environmental factors may also play an important role in AS progression [1,2,3]. To identify each factor that promotes or suppresses AS progression will allow us to better comprehend AS pathogenesis and discover a potential therapy for AS.

Circular RNAs (circRNAs) are single-stranded RNAs with a covalently closed loop structure. CircRNAs are derived from back-splicing of pre-mRNA, in that a covalent linkage of 3′ splice donors with 5′ splice acceptors in the reverse direction [6,7,8,9]. Unlike the linear RNAs, circRNAs are the single-chain closed molecules lacking free 3′-end polyA tail and 5′-end cap, making them more stable and resistant to digestion by RNase R [10]. Recently, many studies have revealed that circRNAs play the critical roles in the regulation of many different human diseases [11,12]. CircRNAs can act as a scaffold molecule, mediating protein–protein interactions to regulate the cellular functions [13,14]. In addition, circRNAs can serve as a sponge to recruit microRNA (miRNA) and interfere with miRNA targeting, in turn affecting the expression of miRNA-targeted genes [8,9]. Many miRNAs, such as miR-27a, miR-29a, miR-335-5p, let-7i, miR-146a, and miR-15, were verified to play a critical role in AS pathogenesis [15,16]. However, there are very few reports to characterize the roles of circRNAs in AS.

Heterogeneous nuclear ribonucleoprotein K (hnRNPK), a member of hnRNPs, acts as a scaffold protein that contains multiple modules for interaction with DNA, RNA, and proteins, allowing it to regulate transcription, RNA metabolism, translation, and signal transduction [17,18]. In addition, hnRNPK also contains one classic nuclear localization signal (NLS) sequence and one nuclear-cytoplasmic shuttling domain at N-terminal region and after the C-terminal K-protein interactive region, respectively, allowing it to be capable of translocation between nucleus and cytoplasm. Moreover, hnRNPK has been found to associate with multiple transcripts in mitochondria [19]. Several lines of evidence have indicated that hnRNPK plays a critical role in the regulation of immune responses [20,21]. hnRNPK modulates inflammatory gene expression by mediating a splicing pattern of transcriptional factors through the hnRNP K-IRF1-CCL5 axis [20]. hnRNPK also plays a critical role in T cell late activation and is required for IL-2 production [21]. hnRNPK can form a complex with VAV1 that is an essential component for T cell activation, in turn protecting VAV1 from proteolysis during T cell activation. hnRNPK in the nucleus is phosphorylated by the activated ERK induced by TCR signaling, promoting its translocation to and accumulation in cytoplasm to enhance the life-time of VAV1 and sustain T cell activation. In this study, we isolated circRNAs from the T cells of AS patients and healthy controls, and used next-generation sequencing (NGS) to analyze their differential expressions of circRNAs between both groups. We characterized one circRNA, circRNPS1, which is up-regulated in T cells of AS patients. Overexpression of circRNPS1 in Jurkat cells promoted the expression of IL-2, IL-17A and interferon-γ (IFN-γ). The mechanism for circRNPS1 to up-regulate cytokine expressions was investigated in this study.

## 2. Results

### 2.1. Differential Expression Profiles of circRNAs in T Cells Isolated from AS Patients and Healthy Controls

**Figure 1:**
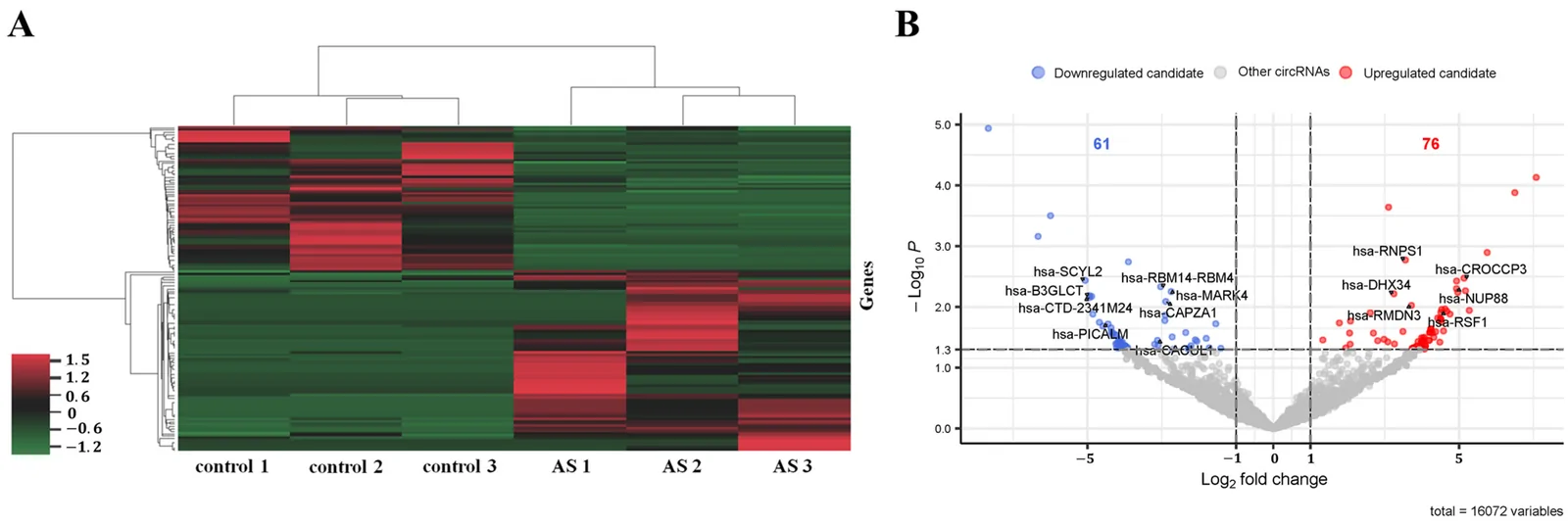
Differential expression profiles of circRNAs in T cells isolated from AS patients and healthy controls. (**A**) Hierarchical clustering analysis of circRNAs meeting the exploratory screening criteria of nominal *p* < 0.05 and |log_2_FC| > 1. (**B**) Volcano plot illustrating circRNA expression differences between the two groups. Red and blue points represent candidate up-regulated and down-regulated circRNAs, respectively, meeting the criteria of nominal *p* < 0.05 and |log_2_FC| > 1. The two vertical dashed lines indicate log_2_FC = +1 and −1, and the horizontal dashed line indicates nominal *p* = 0.05 (−log_10_*p* = 1.30). *p* values were adjusted for multiple comparisons using the Benjamini–Hochberg false discovery rate (FDR) procedure; no circRNA met an FDR threshold of <0.05.

We have systematically examined the differential expression levels of circRNAs between T cells isolated from AS patients and healthy controls respectively by using high-throughput RNA sequencing. The differential expression levels in total circRNAs of T cells between two groups analyzed by high-throughput RNA sequencing with *p* value < 0.05 and >log_2_ fold change are shown in Figure 1A. Compared with the healthy controls, the differential expression levels of circRNAs up-regulated and down-regulated candidates with *p* value < 0.05 and >log_2_ fold change are plotted in Figure 1B. Furthermore, 76 and 61 circRNA candidates in T cells of AS patients are up-regulated and down-regulated, respectively, more than 2 log_2_ fold change with *p* value < 0.05, and are summarized in the Supplementary Tables S1 and S2. Among them, circRNAs for six up-regulated and eight down-regulated candidates are labeled in Figure 1B.

### 2.2. The Levels of circRNPS1 Were Up-Regulated in T Cells of AS Patients

**Figure 2:**
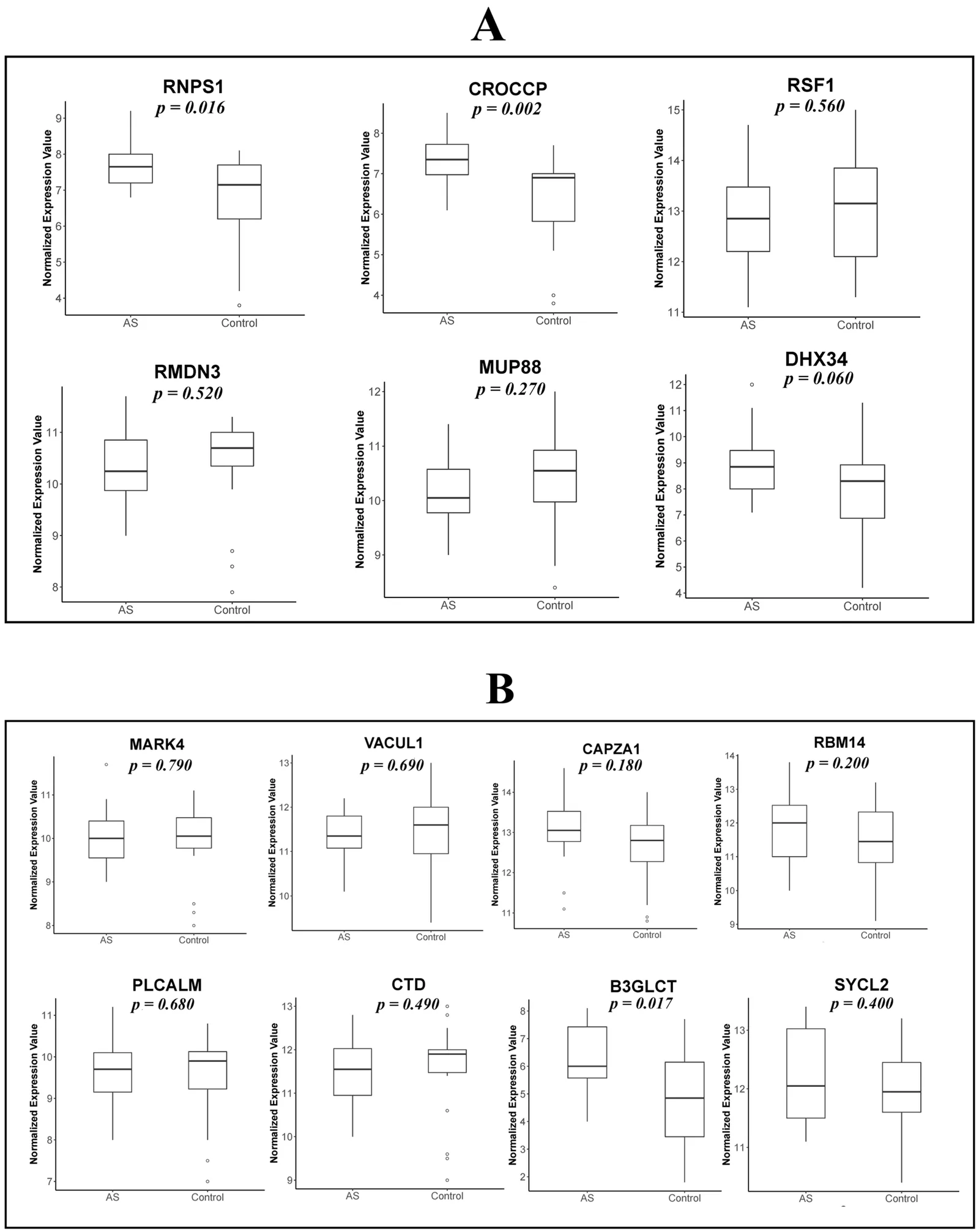
Quantitative real-time PCR. Six up-regulated (**A**) and eight down-regulated circRNA candidates (**B**) were analyzed by qRT-PCR. Relative expression levels of circRNAs were determined by the following equation: (40—threshold cycle [Ct] after adjustment by the expression of GAPDH mRNA). T cells isolated from 20 AS patients and 20 healthy controls were used in assays.

We randomly selected six up-regulated and eight down-regulated circRNA candidates for analysis by quantitative RT-PCR. These selected circRNAs are based on the significant differences in the high number count of back splice junction between AS patients and healthy controls. The names of these circRNAs are shown in the Supplementary Table S3. The expression levels of targeted circRNAs in T cells of AS patients (*n* = 20) or healthy controls (*n* = 20) were analyzed by qRT-PCR using the primers shown in Supplementary Table S4. Compared with the healthy controls, two, circRNPS1 and circCROCCP3, of the six examined circRNAs and one, circB3GLCT, of the eight examined circRNAs were significantly increased (Figure 2A) and decreased (Figure 2B), respectively, in T cells of AS patients after verification by qRT-PCR.

### 2.3. Overexpression of circRNPS1 in Jurkat Cells Up-Regulates the Expression of IL-2, IL-17A, and IFN-γ and Promotes the JAK/STAT1 Signaling in Response to the Treatment with PMA Plus Ionomycin

**Figure 3:**
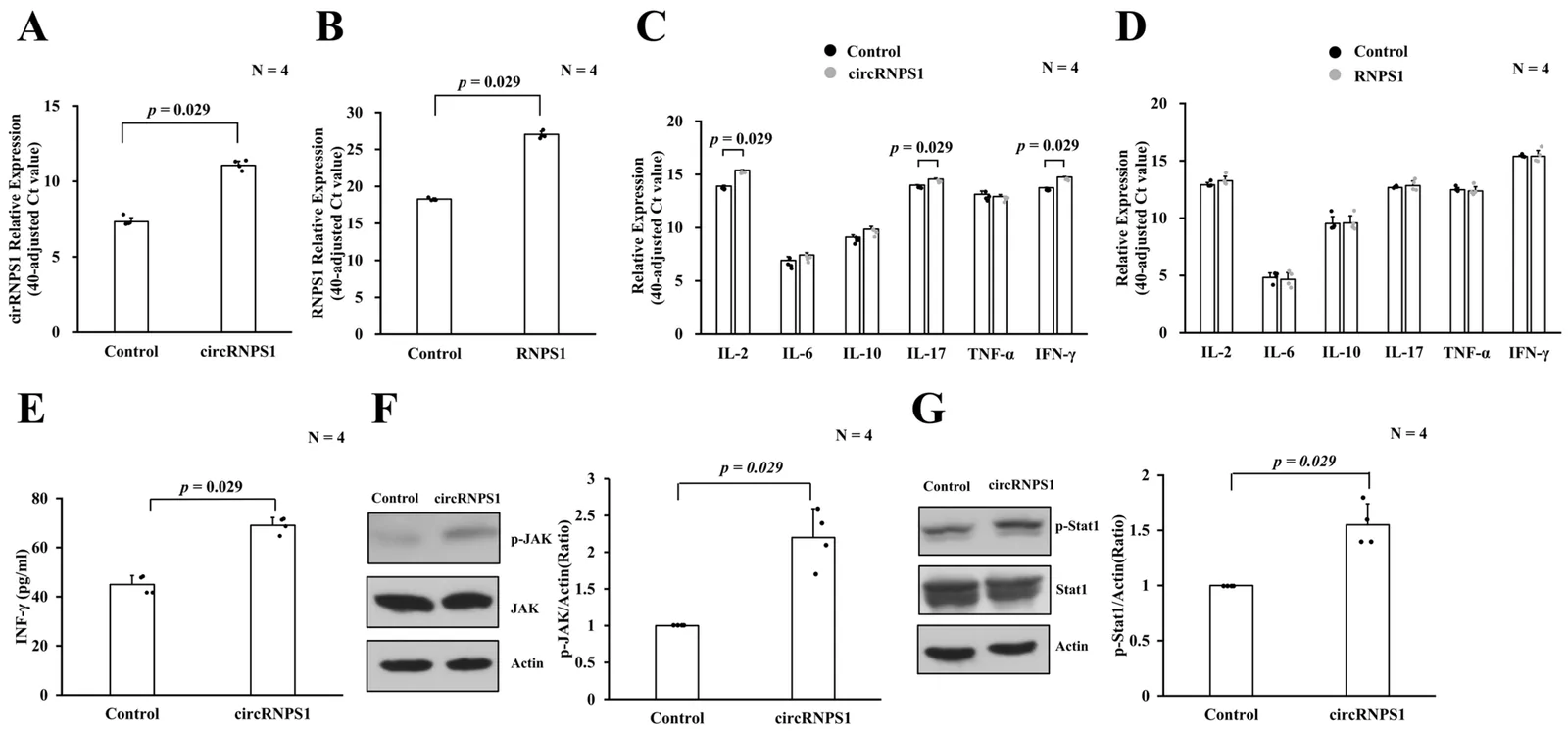
The effect of circRNPS1 on the expression of inflammatory cytokines and on JAK/STAT1 signaling. (**A**) The levels of expressed circRNPS1 analyzed by quantitative RT-PCR. (**B**) The levels of expressed linear RNPS1 analyzed by quantitative RT-PCR. (**C**) The effect of circRNPS1 on the expression of proinflammatory cytokines. (**D**) The effect of linear RNPS1 on the expression of proinflammatory cytokines. (**E**) Quantitation of IFN-γ by ELISA. Jurkat cells (1.5 × 10^6^ cells) were transfected with pcDNA3.1-circRNPS1 (1 μg), pcDNA3.1-linearRNPS1 (1 μg), or the control pcDNA3.1(+) vector (1 μg). The transfected cells were maintained in RPMI-1640 medium with 10% fetal calf serum at 37 °C with 5% CO_2_, PMA (20 ng/mL), and ionomycin (500 ng/mL) for 48 h. IFN-γ released to medium was analyzed by ELISA. (**F**) The effect of circRNPS1 on JAK activation. (**G**) The effect of circRNPS1 on STAT1 activation.

In order to characterize whether circRNPS1 can regulate the inflammatory reactions, the plasmid, pcDNA3.1-circ-RNPS1 or pcDNA3.1-linear-RNPS1, was cloned and overexpressed in Jurkat cells. The mRNA levels of some proinflammatory cytokines were analyzed by using qRT-PCR. Figure 3A,B indicate that circRNPS1 and linear-RNPS1 were overexpressed in Jurkat cells, respectively. In addition, overexpression of circRNPS1 enhances the expression of IL-2. IL-17A and IFN-γ, but does not affect the mRNA expression levels of TNF-α, IL-6, and IL-10 (Figure 3C). However, overexpression of linear-RNPS1 in Jurkat cells does not affect the expression of proinflammatory cytokines, IL-2. IL-17A, TNF-α, IL-6, IL-10, and IFN-γ (Figure 3D). ELISA analysis also confirmed that overexpression of circRNPS1 up-regulates the protein production of IFN-γ (Figure 3E). We asked whether overexpression of circRNPS1 affects the IFN-γ signaling in response to the treatment with PMA plus ionomycin. IFN-γ binds to its receptor and induces the dimerization of its receptor, in turn triggering JAK2/STAT1 signaling [22]. The JAK2/STAT1 signaling is activated by phosphorylation. Therefore, we examined whether overexpression of circRNPS1 in Jurkat cells can promote the levels of phospho-JAK2 and phospho-STAT1 in response to the treatment with PMA plus ionomycin. Overexpression of circRNPS1 in the activated Jurkat cells can up-regulate the phosphorylation of JAK2 and STAT1 (Figure 3F,G), indicating that the signaling of IFN-γ was enhanced after overexpression of circRNPS1.

### 2.4. hnRNPK Binds to the Back-Splicing Junction Site of circRNPS1

**Figure 4:**
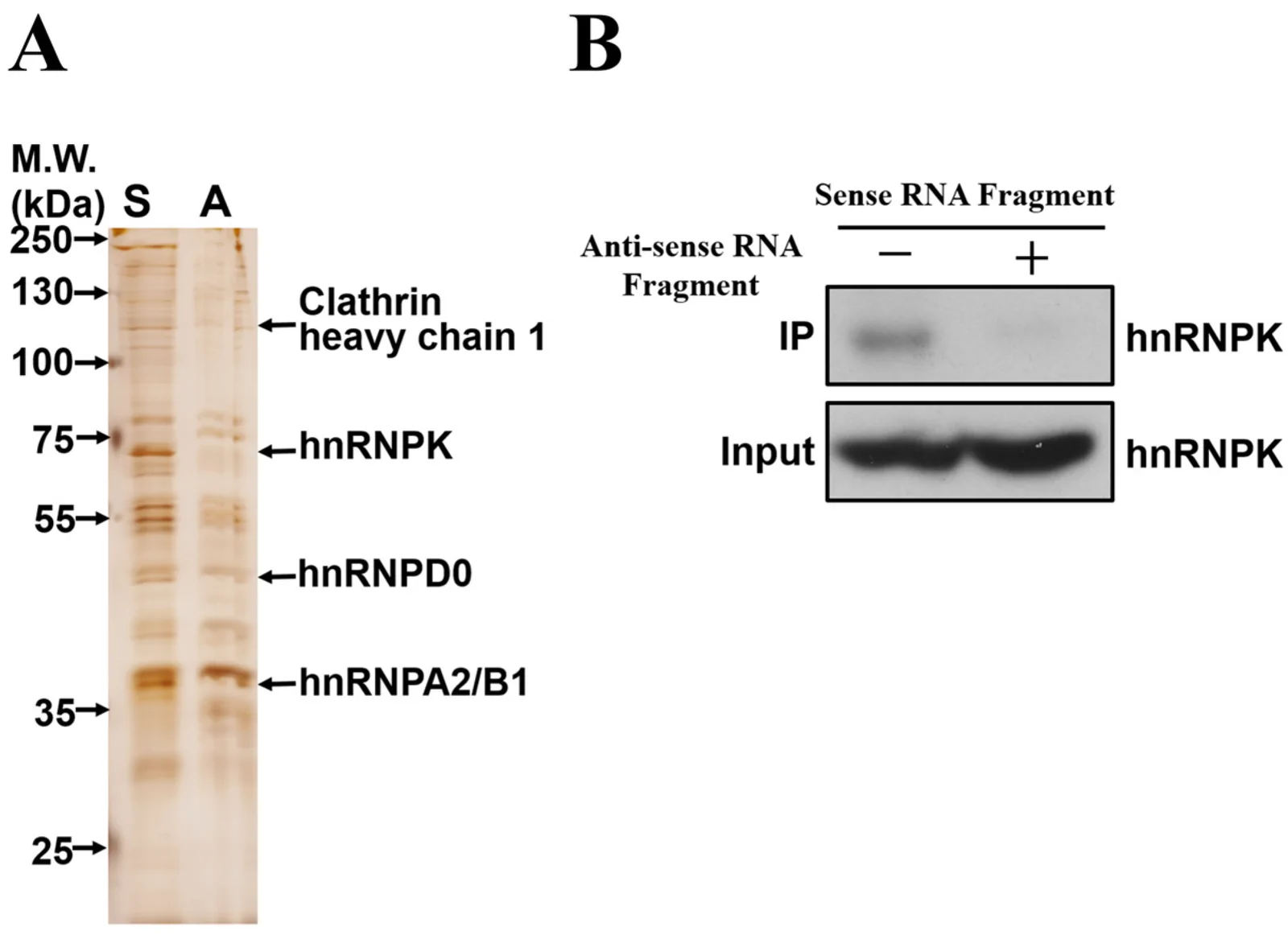
Identification of proteins binding to the back-splicing junction site of circRNPS1. (**A**) Analysis of the proteins binding to the back-splicing junction site of circRNPS1 by SDS-PAGE plus proteomic approaching. Lanes S and A represent the nuclear proteins (1.5 mg) isolated from Jurkat cells incubated with the biotin-labeled sense and antisense RNA fragments (1 μg) that cover the back-splicing junction site of circRNPS1, respectively. The resulting product was precipitated by streptavidin-coupled Dynabeads (60 μL). The arrows represent the proteins subjected to analysis and identification by mass spectrometry and proteomics. (**B**) Western blotting to analyze hnRNPK that was precipitated by RNA pull-down assay.

CircRNAs can function as a scaffold molecule to facilitate RNA–protein interactions, in turn regulating the cellular functions [13,14]. In order to explore the mechanism for circRNPS1 to regulate the expression of cytokines, we carried out the RNA pull-down assay to identify the nucleoproteins associated at the back-splicing junction region of circRNPS1. The precipitated proteins were separated by SDS-PAGE. After silver staining, some targeted proteins on gel were excised and subjected to the analysis by mass spectrometry and proteomics. Figure 4A shows the nucleoproteins that are precipitated by the biotin-labeled sense RNA fragment covering the back-splicing junction region of circRNPS1. Some of the proteins that are only precipitated by the sense RNA fragment are shown in Figure 4A, including clathrin heavy chain 1, nuclear heterogeneous nuclear ribonucleoprotein K (hnRNPK), hnRNP D0, and hnRNP A2/B1. Their proteomic analysis data are shown in Supplementary Table S5. hnRNPK is one of the proteins that binds to the back-splicing junction region of circRNPS1. This interaction is also observed in RNA pull-down assay by the back-splicing junction region of circRNPS1 plus immunoblotting (Figure 4B).

### 2.5. Regulation of MEK1/2-ERK Signaling by circRNPS1 Is an hnRNPK-Dependent Manner

**Figure 5:**
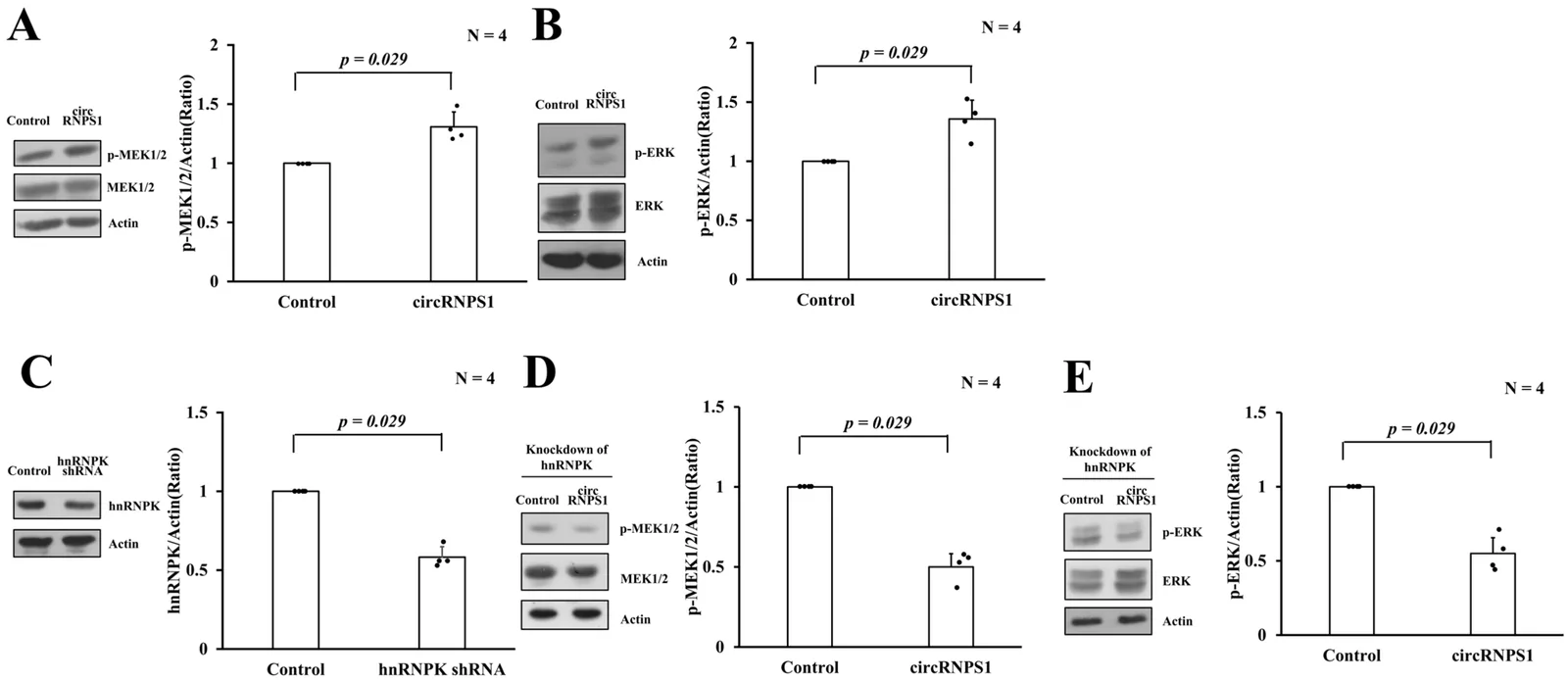
The effect of hnRNPK in the circRNPS1-overexpressed Jurkat cells on the MEK1/2-ERK signaling. (**A**) Overexpression of circRNPS1 in Jurkat cells promotes MEK1/2 activation (*N* = 4). (**B**) Overexpression of circRNPS1 in Jurkat cells promotes ERK activation (*N* = 4). (**C**) Knockdown of hnRNPK by shRNA in the circRNPS1-overexpressed Jurkat cells. The effect of hnRNPK knockdown in circRNPS1-overexpressed Jurkat cells on MEK1/2 (**D**) and ERK (**E**) activation (*N* = 4).

hnRNPK has been known to play a critical role in IL-2 production mediated by sustained ERK signaling in T cell late activation [21]. Thus, we asked whether MEK1/2-ERK signaling is affected by circRNPS1 through hnRNPK. circRNPS1 was overexpressed in Jurkat cells. After the circRNPS1-overexpressed Jurkat cells were treated with PMA plus ionomycin, the activated MEK1/2 and ERK were analyzed by Western blotting. Figure 5A,B show that overexpression of circRNPS1 can increase the phosphorylated levels of MEK1/2 and ERK, respectively, suggesting that MEK1/2-ERK signaling is promoted. When hnRNPK in the circRNPS1-overexpressed Jurkat cells was knocked down, MEK1/2-ERK signaling was suppressed (Figure 5C–E). Taken together, enhancement of MEK1/2-ERK signaling by circRNPS1 is hnRNPK-dependent upon stimulation by PMA and ionomycin.

### 2.6. circRNPS1 Promotes the Stability of hnRNPK and VAV1 as Well as the NF-κB Signaling

**Figure 6:**
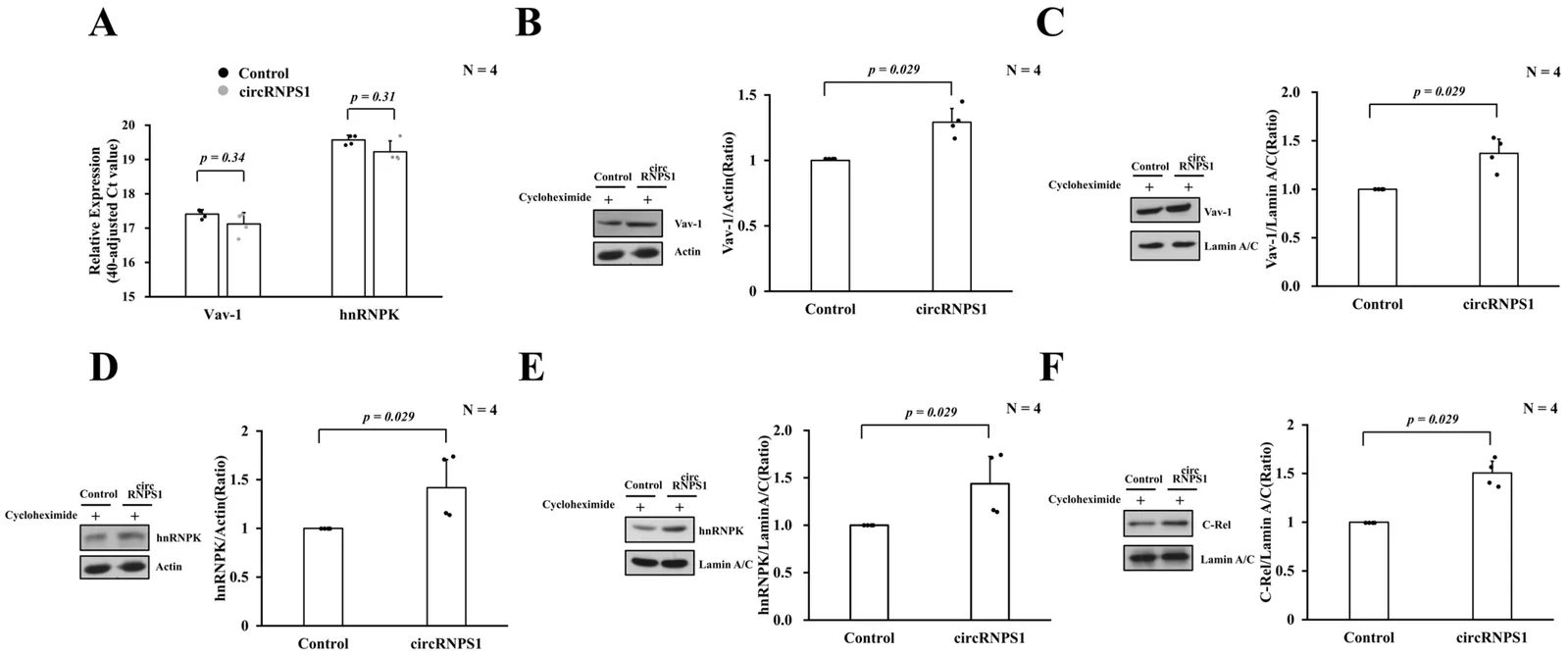
The effect of circRNPS1 overexpression in Jurkat cells on the stability of VAV1 and hnRNPK as well as on NF-κB signaling. The effect of circRNPS1 overexpression on the levels of VAV1 and hnRNPK mRNAs (**A**), on the stability of VAV1 in the cytosol (**B**) and nucleus (**C**), on the stability of hnRNPK in the cytosol (**D**) and nucleus (**E**) and on the levels of c-Rel in nucleus (**F**).

VAV1 is a guanosine nucleotide-exchange factor that catalyzes replacement of bound GDP with GTP for small G proteins of the Rho family [23,24]. VAV1 plays a critical role in the activation of MEK1/2-ERK signaling during T cell activation. hnRNPK can associate with VAV1 and prevent VAV1 from degradation by protease [21]. Thus, we asked whether circRNPS1 affects the transcription of *hnRNPK* and *VAV1*. Jurkat cells with or without overexpression of circRNPS1 were activated by PMA and ionomycin, and the expression levels of hnRNPK and VAV1 mRNA were analyzed by qRT-PCR. Figure 6A indicate that overexpression of circRNPS1 cannot affect transcription of *hnRNPK* and *VAV1*. Then, we examined whether circRNPS1 promotes the MEK1/2-ERK signaling through enhancement of stability of hnRNPK and VAV1. Cycloheximide was added to inhibit the translation during activation of Jurkat cells by PMA and ionomycin. Figure 6B,C show that overexpression of circRNPS1 can increase the stability of VAV1 in cytosol and nucleus, respectively. An increase in stability of hnRNPK is also observed in the cytosol (Figure 6D) and nucleus (Figure 6E) of the circRNPS1-overexpressed Jurkat cells. In addition to the activation of MEK1/2-ERK signaling, hnRNPK and VAV1 can also activate the transcription factors, NF-κB and NFAT [21]. Figure 6F shows that overexpression of circRNPS1 in Jurkat cells can promote the translocation of c-Rel from cytoplasm to nucleus, suggesting that the NF-κB signaling is up-regulated under this condition.

### 2.7. hnRNPK Is Also Involved in the Expressions of IL-17A and IFN-γ in the circRNPS1-Overexpressed Jurkat Cells in Response to the Treatment with PMA and Ionomycin

**Figure 7:**
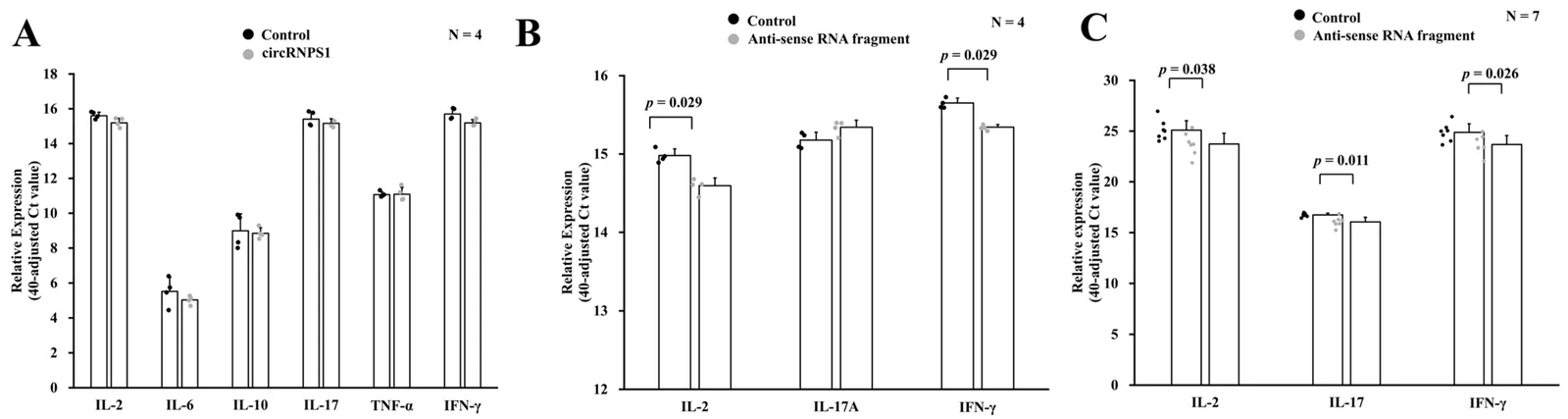
The effect of hnRNPK interacted with circRNPS1 on the proinflammatory cytokine expressions in activated Jurkat cells or in activated T cells. (**A**) The effect of hnRNPK knockdown on the proinflammatory cytokine expressions in the circRNPS1-overexpressed Jurkat cells. (**B**) The effect of transfection with the anti-sense RNA fragment on the expressions of IL-2, IL-17A, and IFN-γ in the circRNPS1-overexpressed Jurkat cells. (**C**) The effect of the anti-sense RNA fragment transfected into T cells isolated from AS patients on the expressions IL-2, IL-17A, and IFN-γ.

Overexpression of circRNPS1 in Jurkat cells also enhances the expression of IL-17A and IFN-γ in response to the treatment with PMA and ionomycin (Figure 3C). We would like to ask whether circRNPS1 also promotes the expression of IL-17A and IFN-γ via hnRNPK. Knockdown of hnRNPK by shRNA was carried out in the circRNPS1-overexpressed Jurkat cells. Figure 7A shows that knockdown of hnRNPK suppresses circRNPS1, enhancing the expression of IL-17A and IFN-γ and suggesting that the hnRNPK/circRNPS1 complex is also involved in regulation of IL-17A and IFN-γ transcriptions in activated Jurkat cells. In addition, transfection of anti-sense RNA sequence that is complementary to the back-splicing junction site of circRNPS1 to block the interaction of hnRNPK with circRNPS1 suppresses the up-regulation of IL-2, IL-17A, and IFN-γ transcriptions induced by circRNPS1 overexpressed in Jurkat cells (Figure 7B). We also isolated T cells from AS patients and transfected the anti-sense RNA fragment into T cells. Although T cells were stimulated by treatment with PMA and ionomycin, the expressions of IL-2, IL-17A, and IFN-γ were suppressed by transfection with anti-sense RNA fragment (Figure 7C).

## 3. Discussion

In this study, we analyzed the differential expressions of circRNAs in T cells between AS patients and healthy controls by using NGS. Compared with the healthy controls, 76 and 61 circRNA candidates in T cells of AS patients are up- and down-regulated, respectively, with more than 2 log_2_ fold change and *p* value < 0.05. We characterized one of the up-regulated circRNAs, circRNPS1, in T cells of AS patients. Overexpression of circRNPS1 can increase the expression of IL-2, IL-17A, and IFN-γ (Figure 3C). RNA pull-down assay and SDS-PAGE plus a proteomic approach demonstrated that clathrin heavy chain 1, hnRNPK, hnRNP D0, and hnRNP A2/B1 bind to the back-splicing junction region of circRNPS1 (Figure 4A).

hnRNPK is a nuclear substrate of TCR-activated ERK [21,25] and is an essential molecule for IL-2 production in T cell late activation [21]. VAV1 has been known to play a critical role in T cell activation and in the activation of ERK signaling [26]. In addition, VAV1 is a component of transcriptional complex of NF-κB and NF-AT [27]. hnRNPK forms a complex with VAV1 in cytosol and nucleus through association of a proline-rich sequence of hnRNPK with the SH3 domain of VAV1 [21,28] and protects VAV1 from proteolysis. These interactions are required for IL-2 production mediated by activated ERK in TCR activation signaling [21]. In this study, we found that hnRNPK binds to the back-splicing junction site of circRNPS1 (Figure 4A,B). This interaction enhances the stability of VAV1 and hnRNPK (Figure 6B–E), leading to overexpression of circRNPS1 and promoting the expression of IL-2 (Figure 3C). In addition, the hnRNPK/circRNPS1 complex also regulates the expression of IL-17A and IFN-γ (Figure 3C and Figure 7A–C), indicating that disruption of hnRNPS1 associated with circRNPS1 suppresses the expression of IL-2, IL-17A, and IFN-γ. Thus, blockage of hnRNPS1 binding to the back-splicing junction site of circRNPS1 is a potential therapeutic targeting site in AS treatment.

Compared with the healthy controls, the expression of circRNPS1 is increased in T cells of AS patients. Overexpression of circRNPS1 in Jurkat cells promotes the expression of IL-2. IL-2 is a pleiotropic cytokine and promotes T cell proliferation and survival. In addition, IL-2 signaling activates the Stat5 transcription factor to induce the expression of *Foxp3* that is essential for differentiation and maintenance of regulatory T (Treg) cells [29,30,31,32]. Conserved noncoding sequences (CNSs) at the *Foxp3* gene locus play a critical role in regulating Fox3 expression. Stat5 activated by the IL-2 signaling binding to the promoter-upstream CNS0 region promotes the *Foxp3* expression. *Foxp3* induction is also controlled by NF-κB signaling mediated by TCR engagement. The active NF-κB directly binds to the promoter, CNS2 and CNS3, within the *Foxp3* gene locus and drives *Foxp3* expression [33,34,35,36,37]. IL-2-Stat5 signaling and TCR activation synergistically enhance *Foxp3* expression, in turn up-regulating the development and maintenance of Treg cells that mediate immune systems to establish and maintain self-antigen tolerance and immune homeostasis as well as prevent the occurrence of autoimmunity [29,30,31,32]. Compared with the healthy controls, expression of IL-2 [38,39] and activity of NF-κB signaling are significantly up-regulated in AS patients, but expression of *Foxp3* and Treg cell development are significantly suppressed in AS patients [40,41]. In our earlier study, we found that the long noncoding RNA, LOC645166, in T cells of AS patients controls the *Foxp3* expression via the axis of LOC645166/miR-188-5p/NFKBID [42]. Compared with the healthy controls, the levels of LOC645166 are down-regulated in T cells of AS patients [42]. LOC645166 serves as a sponge to associate with miR-188-5p, allowing the complex to be degraded and lessening the levels of miR-188-5p. miR-188-5p also pairs with the 3′-UTR of NFKBID mRNA, initiating the degradation of miR-188-5p/NFKBID mRNA and reducing the translation of NFKB1D mRNA. Thus, down-regulation of LOC645166 in T cells of AS patient leads to augmentation of miR-188-5p and in turn reduction in NFKBID expression [42]. NFKBID, also called IκB_ND_, plays a critical role in the regulation of *Foxp3* expression. NFKBID complexed with NF-κB (p50 or c-Rel) binds to the promoter and CNS3 of *Foxp3* locus and triggers the *Foxp3* expression, inducing Treg cell development in thymus [43]. The levels of miR-188-5p and NFKBID protein are increased and decreased in T cells of AS patients, respectively [42]. Therefore, the expression of *Foxp3* in T cells and development of Treg cells are suppressed in AS patients, showing the low levels of CD4^+^CD25^+^FOXP3^+^ Treg cells. In addition, Treg cells in active AS patients display the mal-functions including CNS2 hyperphosphorylation at the *Foxp3* gene locus, the lower levels of IL-2-Stat5 signaling in response to IL-2 stimulation, and being unable to completely suppress naïve T cell proliferation [44].

The molecular mechanism for circRNPS1 to promote the stability of hnRNPK remains unknown. However, the recent study has revealed that stability of hnRNPK is governed by the cullin 3/speckle-type POZ protein (SPOP) complex [45]. Cullin/SPOP, an E3 ubiquitin ligase complex, catalyzes a polyubiquitination reaction on hnRNPK, triggering its degradation by proteosome. Thus, circRNPS1 promotes stability of hnRNPK through formation of a circRNPS1/hnRNPK complex that may prevent hnRNPK from polyubiquitination by the cullin/SPOP complex and suppress degradation of hnRNPK by proteosome.

## 4. Materials and Methods

### 4.1. Isolation of circRNAs from T Cells of AS Patients and Healthy Controls for High-Throughput RNA Sequencing and qRT-PCR

The total RNAs were isolated from T cells of AS patients and healthy controls, respectively, following the methods as described [42,46]. After removal of rRNAs and linear RNAs by digestion with RNase R (1 μg/10U, LGC Biosearch Technologies, Berlin, Germany) at 37 °C for 30 min, the left RNAs were subjected to high-throughput RNA sequencing. For the circRNA sequencing analysis, *p* values obtained from comparisons between AS patients and healthy controls were adjusted for multiple testing across all analyzed circRNAs using the Benjamini–Hochberg procedure. An FDR < 0.05 was considered statistically significant. Nominal *p* < 0.05 together with |log_2_ fold change| > 1 was used only as an exploratory criterion for candidate selection for subsequent qRT-PCR evaluation. For qRT-PCR, 14 targeted circRNAs (1 μg) were analyzed. The targeted circRNAs and primers used for qRT-PCR were summarized in Supplementary Tables S3 and S4, respectively. Relative expression levels of circRNAs were defined by the following equation: (40—threshold cycle [Ct] after being adjusted by the expression of GAPDH).

### 4.2. qRT-PCR Analysis of the Proinflammatory Cytokine, hnRNPK and VAV1 Expressions

The full-length of RNPS1 cDNA was cloned to pcDNA3.1(+) circRNA Mini Vector and pcDNA3.1(+) to generate pcDNA3.1-circRNPS1 and pcDNA3.1-linearRNPS1 that expresses circRNPS1 and linear RNPS1, respectively. Both vectors were ordered from NovoPro Bioscience Inc. (Shanghai, China). Jurkat cells (1.5 × 10^6^ cells) were transfected with the plasmid vector (1 μg) by using electroporation. The transfected cells were maintained in the RPMI-1640 medium with 10% fetal calf serum and 5% CO_2_ at 37 °C for 24 h. The cells were treated with PMA (10 ng/mL) and ionomycin (0.5 μg/mL) for 6 h and then harvested by centrifugation. Isolation of the total RNAs and qRT-PCR analysis of IFN-γ, TNF-α, IL-2, IL-6, IL-17A, or IL-10 mRNA followed the methods as described [47]. Knockdown of hnRNPK in Jurkat cells was carried out using the following method. Jurkat cells (6 × 10^6^ cells) were transfected with 1 μg of hnRNPK shRNA plasmid (catalog #: sc-38282-SH, Santa Cruz Biotechnology, Dallas, TX, USA) by using electroporation. The treated cells were maintained in the RPMI-1640 medium with 10% fetal calf serum, 5% CO_2_, and puromycin (0.3 μg/mL) at 37 °C for selection of stable clone. To examine the effect of hnRNPK knockdown on the proinflammatory cytokine expressions in the circRNPS1-overexpressed Jurkat cells, Jurkat cells (1.5 × 10^6^ cells) with knockdown of hnRNPK were transfected with pcDNA3.1-circRNPS1 (1 μg) or pcDNA3.1-linear-RNPS1 (1 μg) by using electroporation. To examine the effect of anti-sense RNA fragment on the expressions of proinflammatory cytokines, the circRNPS1-overexpressed Jurkat cells (1.5 × 10^6^ cells) or T cells (1.5 × 10^6^ cells) isolated from AS patients were transfected with the anti-sense RNA fragment (1 μg) or the scrambled control RNA fragment (1 μg) by electroporation. The sequence of anti-sense RNA fragment (5′-biotin-aaaCCT CGG GGA GCG GGA CAC CAC CAT GAA CGG CAT-3′) is complementary to the region covering the back-splicing junction site of circRNPS1. Cell activation by PMA and ionomycin as well as qRT-PCR analysis of the proinflammatory cytokine mRNAs followed the above-mentioned method. qRT-PCR analysis of hnRNPK or VAV1 was carried out by using the primers (5′-GGG GGA TCA TGC CCT TCC C-3′/5′-TCG TCG CCT TCC GCC TCC-3 for VAV1; 5′-CCC AGA CAG CAG TGG CCC-3′/5′-GAT TCG AGC GGG AGC TGG C-3′ for hnRNPK). The relative expression levels of mRNA were defined by the following equation: (40—threshold cycle [Ct] after being adjusted by the expression of 18S rRNA).

### 4.3. circRNPS1 Pull-Down Assay

The sequences 5′-biotin-aaaTGC CGT TCA TGG TGG TGT CCC GCT CCC CGA GG-3′ and 5′-biotin-aaaCCT CGG GGA GCG GGA CAC CAC CAT GAA CGG CAT-3′ are the biotin-labeled sense and anti-sense oligomers that cover the back-splicing junction site of circRNPS1, respectively, both of which were ordered from Mission Biotech Co. (Taipei, Taiwan). The back-splicing junction site is marked in bold. Jurkat cells (2.5 × 10^6^ cells) were seeded into 5 cm Petri dishes and maintained in RPMI-1640 medium with 10% FBS and 1% penicillin/streptomycin. After 16 h, the cells were harvested by centrifugation at 1200× *g* for 5 min. Subcellular fractionation as well as extraction of the cytoplasmic and nuclear proteins followed the methods as described [48]. For the pull-down of nuclear proteins, biotinylated RNA oligomer (1 μg) was mixed with 1.5 mg of the extracted nuclear proteins resuspended in the IP buffer (150 mM NaCl, 20 mM Tris-HCl (pH 7.4), 1 mM EDTA, 0.5% Triton X-100 with protease inhibitors (Millipore, Burlington, MA, USA) and RNaseOUT supplemented with 0.2 mg/mL heparin, 0.2 mg/mL yeast tRNA, and 1 mM dithiothreitol) [49]. All components were allowed to stand at 4 °C for two hours, followed by the addition of the washed streptavidin-coupled Dynabeads (60 μL). After incubation at 4 °C for two hours, the precipitates were washed four times with the IP buffer and collected by centrifugation (760× *g*) at 4 °C for 2 min. The precipitated products were subjected to the analysis by SDS-PAGE, followed by silver staining. The target molecules on gel were excised and digested by trypsin. The tryptic fragments were analyzed by mass spectrometry and proteomics.

### 4.4. Western Blotting

Jurkat cells (1.5 × 10^6^ cells) or Jurkat cells with knockdown of hnRNPK (1.5 × 10^6^ cells) were transfected with pcDNA3.1-circRNPS1or pcDNA3.1-linear RNPS1, following the above-mentioned method. Cells were maintained in the RPMI-1640 medium with 10% fetal calf serum at 37 °C with 5% CO_2_ for 24 h, followed by the treatment with PMA (10 ng/mL) and ionomycin (0.5 μg/mL) for 6 h. The cells were harvested by centrifugation, resuspended in 100 μL of 1% SDS, ruptured by ultrasonication, and analyzed by Western blotting, probed for actin, STAT1, phospho-STAT1, JAK2, phospho-JAK2, MEK1/2, phospho-MEK1/2, ERK, or phospho-ERK.

### 4.5. Measurement of Protein Stability

Jurkat cells (2 × 10^5^ cells) with or without stable overexpression of circRNPS1 were seeded into 6 cm Petri dishes and maintained in the RPMI-1640 medium with 10% FBS and 1% penicillin/streptomycin at 37 °C with 5% CO_2_. After 24 h, the cells were treated with cycloheximide (100 μg/mL) and stimulated with PMA (10 ng/mL) and ionomycin (0.5 μg/mL). After 6 h, the cells were harvested by centrifugation at 1500× *g* for 5 min. Subcellular fractionation followed the above-mentioned method. Aliquots of cytoplasmic and nuclear proteins were analyzed by SDS-PAGE, respectively, and Western blotting, probed for hnRNPK and VAV1. The cytoplasmic and nuclear fractions were identified with anti-actin and anti-lamin antibodies, respectively.

### 4.6. ELISA

Determination of IFN-γ concentration in the culture medium used the kit from BD Biosciences (Catalog No. 550612, Franklin Lakes, NJ, USA), following the methods described by the protocol of the manufacturer.

### 4.7. Mass Spectrometric Analysis

The targeted bands on gel were excised and washed with 0.1% trifluoroacetic/50% acetonitrile to remove the excess reagents. The resulting gels were dried by vacuum centrifugation, followed by re-suspension in 50 mM ammonium bicarbonate, reduction with 10 mM dithiothreitol at 60 °C for 45 min, and then reaction with 55 mM iodoacetamide at 25 °C for 30 min to block the thiol group of cysteinyl residues. The resulting gels were treated with trypsin at 37 °C for 16 h. The released tryptic peptides were then extracted from gel, dried by vacuum centrifugation, and resuspended with 0.1% formic acid. The tryptic peptides were diluted in HPLC buffer A (0.1% formic acid) and loaded onto a reverse-phase column (Zorbax 300SB-C18, 0.3 × 5 mm; Agilent Technologies, Santa Clara, CA, USA). The desalted peptides were then chromatographed on a home-packed column (Waters BEH C18 resin with dimensions of 1.7 µm particle size; 100 μm I.D. × 10 cm with a 15 μm electrospray emitter tip) using a multi-step gradient of HPLC buffer B (99.9% acetonitrile/0.1% formic acid) for 70 min with a flow rate of 0.3 μL/min. The LC instrument coupled with a 2D linear ion trap mass spectrometer (Orbitrap Elite ETD; Thermo Fisher, Waltham, MA, USA) was operated using Xcalibur 2.2 software (Thermo Fisher). Protein identification was carried out using Proteome Discoverer software (version 2.3, Thermo Fisher Scientific). The MS/MS spectra were searched against the customized database using the Mascot search engine (Matrix Science, London, UK; version 2.5). Mass spectrometric analysis was carried out by OmicsLab Co. (New Taipei City, Taiwan).

### 4.8. Statistical Analysis

Data are presented as means ± SD. Statistical significance was analyzed by the Mann–Whitney U test. *p* values < 0.05 were considered statistically significant. Statistical analyses were performed using IBM SPSS for Windows, Version 22.0 (IBM Corp., Armonk, NY, USA).

## Data Availability

The datasets shown in this study are available from the corresponding authors on reasonable request.

## Supplementary Materials

The following supporting information can be downloaded at https://www.mdpi.com/article/10.3390/ijms27177550/s1.

## Abbreviations

AS	Ankylosing spondylitis
CCL5	Chemokine (C-C motif) ligand 5
circRNA	Circular RNA
ELISA	Enzyme-linked immunosorbent assay
ERK	Extracellular signal-regulated kinase
HLA	Human leukocyte antigen
hnRNP	Heterogeneous nuclear ribonucleoprotein
IFN-γ	Interferon-γ
IL	Interleukin
IRF1	Interferon regulatory factor 1
JAK	Janus kinase
MEK	Mitogen-activated protein kinase kinase
NFAT	Nuclear factor of activated T cells
NF-κB	Nuclear factor kappa-light-chain-enhancer of activated B cells
PMA	Phorbol 12-myristate 13-acetate
qPT-PCR	Quantitative real-time reverse transcription-polymerase chain reaction
RNPS1	RNA-binding protein with serine-rich domain 1
SDS-PAGE	Sodium dodecyl sulfate polyacrylamide gel electrophoresis
SPOP	Speckle-type POZ protein
STAT	Signal transducer and transcription activator
TCR	T cell receptor
