# Supplementary material for: An Increased Circular RNA, circRNPS1, in T Cells of Ankylosing Spondylitis Patients Enhances the MEK/ERK Signaling and Inflammation Mediated by T Cell Activation via Stabilization of hnRNPK and VAV1
Source: Int J Mol Sci. 2026 Aug 23;27(17):7550. doi: 10.3390/ijms27177550 (PMC13566842; doi:10.3390/ijms27177550)
Supplement: Supplementary file 1 [file ijms-27-07550-s001.zip › ijms-4410438-supplementary.pdf]

An Increased Circular RNA, circRNPS1, in T Cells of Ankylosing Spondylitis Patients Enhances the MEK/ERK Signaling and Inflammation Mediated by T Cell Activation via Stabilization of hnRNPK and VAV1

Hui-Chun Yu [1,†], Ning-Sheng Lai [2,3,†], Kuang-Yung Huang [2,3], Pin-Chen Chen [1], Ming-Chi Lu [1,2,3,*] and Hsien-Bin Huang [4,*]

[1] Department of Medical Research, Dalin Tzu Chi Hospital, Buddhist Tzu Chi Medical Foundation, Chiayi 62247, Taiwan; junvsusagi@gmail.com (H.-C.Y.); love19961215@gmail.com (P.-C.C.)
[2] Division of Allergy, Immunology and Rheumatology, Department of Medicine, Dalin Tzu Chi Hospital, Buddhist Tzu Chi Medical Foundation, Chiayi 62247, Taiwan; q12015@tzuchi.com.tw (N.-S.L.);
hky0919@yahoo.com.tw (K.-Y.H.)
[3] School of Medicine, Tzu Chi University, Hualien 62102, Taiwan
[4] Department of Biomedical Sciences, National Chung Cheng University, Chiayi 62102, Taiwan
* Correspondence: dm252940@tzuchi.com.tw (M.-C.L.); biohbh@ccu.edu.tw (H.-B.H.)
† These authors contributed equally to this work.

Supplementary Table S1. Seventy-six up-regulated circRNA candidates in T cells of AS patients.

circRNA ID	Number of back splice junction					
	AS1	AS2	AS3	control1	control2	control3
6:29890264|29944616	0	0	33	0	0	0
17:10495192|10630458	0	59	0	0	0	0
Y:19587210|19587507	25	34	14	1	0	13
5:21491321|21495182	26	0	0	0	0	0
16:2253304|2254063	9	17	2	0	2	0
1:16486426|16486661	2	4	5	0	0	0
7:100338179|100338889	7	8	0	0	0	0
17:5410703|5414134	4	3	3	0	0	0
5:21491321|21497196	18	0	0	0	0	0
3:195959183|195960086	0	21	0	0	0	0
19:47362476|47362693	6	8	4	0	2	0
15:40744047|40745259	4	5	4	1	0	0
10:45939361|45962994	9	3	0	0	0	0
21:42850041|42876767	5	3	1	0	0	0
6:57926426|57930291	0	0	11	0	0	0
3:138570318|138572932	0	7	3	0	0	0
11:77683710|77685159	2	4	2	0	0	0
17:12054889|12081530	9	6	7	0	6	0
5:176943335|176951577	4	0	4	0	0	0
12:14446988|14457295	1	5	2	0	0	0
16:50786855|50793664	0	8	2	0	0	0
7:72890627|72891110	0	16	0	0	0	0
5:39153448|39159920	2	2	3	0	0	0
17:66513336|66514466	20	23	4	2	0	12
22:40881770|40882177	6	0	2	0	0	0
10:7797047|7802854	17	48	10	14	3	6
19:1147309|1154402	4	2	1	0	0	0
5:172117481|172156788	4	2	1	0	0	0
8:108449823|108450492	4	2	1	0	0	0
12:68654112|68654252	1	0	6	0	0	0
5:179841907|179844227	3	3	4	1	0	0
7:128574720|128580094	5	0	2	0	0	0
3:150562542|150563614	2	2	2	0	0	0
15:90486954|90492711	5	1	1	0	0	0
17:82905936|82911789	5	1	1	0	0	0
12:94209590|94227235	1	2	3	0	0	0
16:14997753|14998405	16	3	14	0	4	4
12:28225795|28226199	3	3	10	0	3	0
17:45237281|45239065	3	0	3	0	0	0
16:30489082|30489386	3	2	1	0	0	0
3:195708134|195708841	3	5	0	0	0	0
3:149895473|149921227	2	3	1	0	0	0
5:108341927|108348530	7	0	6	0	2	0
14:96520055|96525391	4	1	1	0	0	0
15:100564692|100565265	66	78	48	64	12	26
22:45700282|45729590	4	0	2	0	0	0
2:18919213|18943902	4	0	2	0	0	0
11:77685105|77693611	4	2	6	3	0	0
20:36578741|36578966	2	1	2	0	0	0
7:149074386|149080717	2	1	2	0	0	0
2:111863675|111863895	2	6	0	0	0	0
11:118583809|118584644	8	6	0	0	0	1
3:101585437|101590270	0	0	7	0	0	0
12:101780152|101780621	1	2	2	0	0	0
16:58582793|58583182	1	2	2	0	0	0
1:217610321|217614202	1	2	2	0	0	0
5:154871730|154872651	1	2	2	0	0	0
6:36902221|36902434	1	2	2	0	0	0
X:101018971|101019403	1	2	2	0	0	0
3:56669700|56673725	2	4	3	1	0	0
19:39329000|39333126	6	5	8	4	0	2
16:66727688|66729099	2	0	3	0	0	0
16:89892193|89896083	2	0	3	0	0	0
3:47867246|47872100	2	0	3	0	0	0
17:5365122|5368468	0	4	2	0	0	0
9:93476256|93498886	3	1	1	0	0	0
16:11020193|11126146	4	3	0	0	0	0
16:66813564|66813846	3	0	2	0	0	0
2:32298234|32301495	3	0	2	0	0	0
1:1256992|1257310	1	3	1	0	0	0
2:32491425|32493667	1	3	1	0	0	0
6:17665239|17669546	1	3	1	0	0	0
10:97437191|97437750	5	6	9	5	1	1
17:78079395|78093763	1	7	0	0	0	0
15:65696366|65697383	0	6	1	0	0	0
4:56478379|56483270	1	0	4	0	0	0

Supplementary Table S2. Sixty-two down-regulated circRNA candidates in T cells of AS patients.

circRNA ID	Number of back splice junction					
	AS1	AS2	AS3	control1	control2	control3
15:58912563|58916999	12	15	3	38	24	20
2:201145378|201149835	8	29	10	47	29	62
3:107710452|107716849	3	6	1	10	7	16
1:1804419|1817875	1	3	1	7	9	7
9:96458379|96465778	0	2	1	6	5	5
9:96458379|96458541	7	0	5	16	13	14
19:45263113|45263367	1	0	3	13	9	9
5:172055588|172057473	6	1	0	10	12	4
3:136260479|136262065	1	2	0	12	6	2
10:68393138|68394451	1	1	0	5	6	4
2:44209210|44218537	6	0	0	8	6	9
1:21715389|21721764	2	0	0	1	4	8
2:147896301|147899898	2	0	0	8	1	13
10:118729295|118730410	1	0	0	9	3	2
7:131052425|131053465	1	0	0	2	7	2
16:57164001|57173869	0	6	0	13	10	2
8:37877109|37877551	0	3	0	9	2	9
11:66639700|66640123	0	5	0	3	6	19
3:52737586|52741499	0	1	0	6	4	2
1:112653598|112659779	0	0	3	8	7	9
14:65455621|65458207	0	0	1	9	5	8
9:83679775|83686155	0	0	0	3	2	1
10:60084602|60085253	0	0	0	1	2	2
2:171046174|171050174	0	0	0	1	2	2
18:9583117|9595153	0	0	0	3	1	2
8:38347497|38348215	0	0	0	3	1	2
X:10063445|10098579	0	0	0	3	1	2
11:36393846|36419303	0	0	0	2	3	1
1:10095461|10105744	0	0	0	4	4	0
15:89888521|89889140	0	0	0	4	0	3
12:46239679|46254936	0	0	0	4	2	1
6:156829227|156829421	0	0	0	2	6	0
5:129104956|129107062	0	0	0	2	1	3
10:70217812|70218817	0	0	0	2	2	2
12:57395749|57398202	0	0	0	7	0	2
16:11846501|11847806	0	0	0	7	0	2
1:213117317|213129889	0	0	0	3	3	1
1:231818329|231819150	0	0	0	1	3	2
7:5216563|5217187	0	0	0	0	1	5
5:56111118|56116782	0	0	0	0	6	1
10:45975454|45988847	0	0	0	5	0	3
6:108928088|108936999	0	0	0	2	0	5
22:22900976|22905025	0	0	0	0	9	0
12:103263326|103270308	0	0	0	5	1	2
2:208345111|208348023	0	0	0	0	2	4
17:60679453|60709325	0	0	0	3	2	2
12:26982773|26990627	0	0	0	2	3	2
14:52757319|52759754	0	0	0	2	1	4
12:28362438|28391411	0	0	0	2	2	3
11:61366045|61367998	0	0	0	4	2	2
11:85974708|85983973	0	0	0	3	3	2
3:67518247|67529186	0	0	0	3	0	6
6:32520164|32580856	0	0	0	21	0	0
6:118448404|118449870	0	0	0	0	3	6
18:47865059|47896809	0	0	0	10	1	3
14:86028369|86041833	0	0	0	6	1	5
13:31215051|31229294	0	0	0	3	5	3
12:100282943|100298175	0	0	0	8	3	3
12:68650400|68654252	0	0	0	0	7	12
21:36338774|36344707	0	0	0	0	0	24
6:31271073|31355592	0	0	0	0	81	0

Supplementary Table S3. The selected circRNAs analyzed by quantitative RT-PCR.

Up-regulation		Down-regulation	
**Name**	**Location**	**Name**	**Location**
hsa-RNPS1	16:2253304丨2254063	hsa-MARK4	19:45263113丨45263367
hsa-CROCCP3	1:16486426丨16486661	hsa-CACUL1	10:118729295丨118730410
hsa-NUP88	17:5410703丨5414134	hsa-RBM14-RBM4	11:66639700丨66640123
hsa-DHX34	19:47362476丨47362693	hsa-CAPZA1	1:112653598丨112659779
hsa-RMDN3	15:40744047丨40745259	hsa-PICALM	11:85974708丨85983973
hsa-RSF1	11:77683710丨77685159	hsa-CTD-2341M24	14:86028369丨86041833
		hsa-B3GLCT	13:31215051丨31229294
		hsa-SCYL2	12:100282943丨100298175

Supplementary Table S4. The primers used for quantitative RT-PCR.

Name	Sequence
circRNPS1-F	GCGGAGTCATCACGCAGCC
circRNPS1-R	AGCTGGAGCGGCTCCTGTG
circCROCCP-F	GACCTGGACAAGGCTGACC
circCROCCP-R	ACAGGCCCGTGCTCAGTCC
circNUP88-F	AGCCGCAGACACCCACTAAC
circNUP88-R	AGTCAGAGAGGTGGAACTGG
circDHX34-F	CGGAGGGTGGCCCTGGAC
circDHX34-R	GGCCCTTCCGCTGCTCTG
circRMDN3-F	CACTGAGGAGGTGAGCGAG
circRMDN3-R	ACACGTCACTTCATCTTCCCC
circRSF1-F	AGGAGGTCAACAAGAACAAGG
circRSF1-R	CAAGCAAGTTTGCTTTGGATTT
circMARK4-F	GCTGTTCCGAGAAGTCCGC
circMARK4-R	CTGTGCCGGAAGCCTGGC
circCACUL1-F	ACTAATCACTTAGAGAGAGTCTC
circCACUL1-R	GGGGATATAGTCACCAGGAC
circRBM14-F	ATGCAGTGGAGGCCATCAG
circRBM14-R	GTCACATTCCAGCAGCTTCC
circCAPZA1-F	CCATCACACCACCTACAGCC
circCAPZA1-R	TCTGCTATTACCCAGGTCACC
circFUT8-F	GGGCGACAGAGCGAGACTCCG
circFUT8-R	GCCCGGCCTCAGTACCTG
circPICALM-F	CAGCCAGCCTGTCATGAGAC
circPICALM-R	GCCCCCAGAATCATCAATAAC
circCTD-F	CTGAGAACATGCTTGATGAAG
circCTD-R	GGCTGCACACTGAGGGCC
circB3GLCT-F	CATGCAAAGAGAGCAGAGCAG
circB3GLCT-R	CTGCCTTCTGGATGCCACAG
circSCYL2-F	TCTACCTTCCCCTATATCTCC
circSCYL2-R	CAGCACTAGTGACATCAGCTG
GAPDH-F	CCATCACCATCTTCCAGGAGCGA
GAPDH-R	GGTGGTGAAGACGCCAGTGGA

Supplementary Table S5. Proteomic analysis of the targeted proteins.

Accession	Description	Coverage [%]	MW [kDa]	Score Mascot: Mascot
Q00610	Clathrin heavy chain 1	50	191.5	13411
P61978	Heterogeneous nuclear ribonucleoprotein K	71	50.9	21605
Q14103	Heterogeneous nuclear ribonucleoprotein D0	45	38.4	4581
P22626	Heterogeneous nuclear ribonucleoproteins A2/B1	56	37.4	4768

Supplementary Table S6. The basic characteristics of the AS patients.

Characteristics	All patients with AS
Patients (n)	27
Age (years), mean ± SD	48.3 ± 9.9
Sex (Male/Female)	17/10
HLA-B27 Positive, n (%)	27(100)
ESR(mm/h), mean ± SD	14.4 ± 9.2
CRP(mg/dl), mean ± SD	0.59 ± 0.96
Anti-TNF-α treatment, n (%)	15 (55.6)
BASDAI	3.07 ± 1.83
